# Impact of mutations in Toll-like receptor pathway genes on esophageal carcinogenesis

**DOI:** 10.1371/journal.pgen.1006808

**Published:** 2017-05-22

**Authors:** Daffolyn Rachael Fels Elliott, Juliane Perner, Xiaodun Li, Martyn F. Symmons, Brett Verstak, Matthew Eldridge, Lawrence Bower, Maria O’Donovan, Nick J. Gay, the OCCAMS Consortium, Rebecca C. Fitzgerald

**Affiliations:** 1 MRC Cancer Unit, Hutchison/MRC Research Centre, University of Cambridge, Cambridge, United Kingdom; 2 Cancer Research UK Cambridge Institute, University of Cambridge, Cambridge, United Kingdom; 3 Department of Biochemistry, University of Cambridge, Cambridge, United Kingdom; 4 Department of Histopathology, Cambridge University Hospital NHS Trust, Cambridge, United Kingdom; Novartis, UNITED STATES

## Abstract

Esophageal adenocarcinoma (EAC) develops in an inflammatory microenvironment with reduced microbial diversity, but mechanisms for these influences remain poorly characterized. We hypothesized that mutations targeting the Toll-like receptor (TLR) pathway could disrupt innate immune signaling and promote a microenvironment that favors tumorigenesis. Through interrogating whole genome sequencing data from 171 EAC patients, we showed that non-synonymous mutations collectively affect the TLR pathway in 25/171 (14.6%, PathScan p = 8.7x10^-5^) tumors. TLR mutant cases were associated with more proximal tumors and metastatic disease, indicating possible clinical significance of these mutations. Only rare mutations were identified in adjacent Barrett’s esophagus samples. We validated our findings in an external EAC dataset with non-synonymous TLR pathway mutations in 33/149 (22.1%, PathScan p = 0.05) tumors, and in other solid tumor types exposed to microbiomes in the COSMIC database (10,318 samples), including uterine endometrioid carcinoma (188/320, 58.8%), cutaneous melanoma (377/988, 38.2%), colorectal adenocarcinoma (402/1519, 26.5%), and stomach adenocarcinoma (151/579, 26.1%). *TLR4* was the most frequently mutated gene with eleven mutations in 10/171 (5.8%) of EAC tumors. The *TLR4* mutants E439G, S570I, F703C and R787H were confirmed to have impaired reactivity to bacterial lipopolysaccharide with marked reductions in signaling by luciferase reporter assays. Overall, our findings show that TLR pathway genes are recurrently mutated in EAC, and *TLR4* mutations have decreased responsiveness to bacterial lipopolysaccharide and may play a role in disease pathogenesis in a subset of patients.

## Author summary

Esophageal adenocarcinoma (EAC) is a deadly human cancer that develops in the lower esophagus, which is exposed to reflux of acidic stomach contents and has reduced microbial diversity. Next generation sequencing studies have shown that accumulation of somatic mutations occurs along the metaplasia–dysplasia–carcinoma sequence in EAC, and it has been proposed that groups of genes involved in similar processes or pathways could have a cumulative effect. The Toll-like receptor (TLR) signalling pathway is critical for host-microbe interaction, and therefore mutations in these pathways could be important in the pathogenesis of EAC. Here we show that TLR pathway genes are recurrently mutated in 15% of EAC tumours and other solid tumour types exposed to microbial communities, including uterine endometrioid carcinoma (59%), cutaneous melanoma (38%), colorectal adenocarcinoma (27%), and stomach adenocarcinoma (26%). TLR mutant cases were associated with more proximal tumours and metastatic disease, indicating possible clinical significance of these mutations. A better understanding of how altered TLR signalling contributes to the inflammatory tumour microenvironment in EAC could help inform cancer prevention strategies.

## Introduction

Esophageal adenocarcinoma (EAC) is increasing in incidence and has poor survival outcomes. The main risk factor for EAC is Barrett’s esophagus, a pre-malignant glandular epithelium that develops in the setting of gastro-esophageal reflux disease. Over time exposure to refluxed acid and bile in the lower esophagus leads to chronic inflammation, increased cell turnover, production of reactive oxygen species and DNA damage [1,2]. The combination of exposure to noxious substances and defects in DNA damage repair enable cancer cells to accumulate mutations, evidenced by characteristic mutational signatures [3,4].

Next generation sequencing studies have shown that accumulation of somatic mutations occurs along the metaplasia–dysplasia–carcinoma sequence in EAC [5,6] and potentially impair important cell functions. However, few genes are mutated in greater than 10% of cases, underlining the heterogeneous nature of point mutations and small indels in this type of cancer. As a result it is difficult to pinpoint the genes that are relevant to tumorigenesis using traditional approaches based on the frequency of mutations in a single gene. It has been proposed that groups of genes involved in similar processes or pathways could have a cumulative effect. An example is the SWI/SNF nucleosome remodeling complex (ARID1A, SMARCA4 and ARID2), for which gene members are mutated collectively in 20% of EAC tumors [6]. Computational tools such as PathScan [7] have been developed to identify cellular pathways that are targeted by somatic mutations above the background mutation rate.

Epidemiologic evidence has linked the rising incidence of EAC with the eradication of *Helicobacter pylori* [8,9], and studies have suggested that the esophageal microbiota are altered in Barrett’s esophagus [10–12] and EAC with decreased microbial diversity [13]. The Toll-like receptor (TLR) signaling pathway is a key component of the innate immune system and one of the main ways in which tumor cells interact with the microbiota. TLRs are pattern recognition receptors that bind unique molecular components of the microbiota and generate a pro-inflammatory innate immune reaction through nuclear factor kappa B (NF-κB) [14]. TLR signaling exerts an effect on epithelial cell function in the gastrointestinal tract, including stimulating repair of damaged enterocytes (lipopolysaccharide), enhancing cell proliferation, and triggering secretion of antimicrobial peptides (lipopolysaccharide and flagellin) [15]. Alongside TLRs, inflammasomes also contribute to inflammation through recognition of pathogen-associated molecular patterns. NLRP6 is a member of the NOD-like receptor family that plays a role in regulating inflammation and epithelial cell repair in the intestine and has been implicated in colorectal carcinogenesis [16,17].

Genome-wide association studies [18,19] and next-generation sequencing studies [5,6] have identified *TLR4* gene mutations in solid tumors including EAC. We hypothesized that somatic mutations may collectively target the TLR signaling pathway in EAC and alter inflammatory signaling. We aimed to interrogate TLR pathway mutations and expression in a cohort of EAC and Barrett’s esophagus patients with clinical outcome data. We then investigated whether *TLR4* mutations in EAC affect downstream inflammatory signaling using an *in vitro* model system. Finally, we aimed to determine the broader relevance of TLR pathway mutations in other cancers using TCGA data and the COSMIC database.

## Results

### The TLR pathway is significantly mutated in EAC

**Fig 1:**
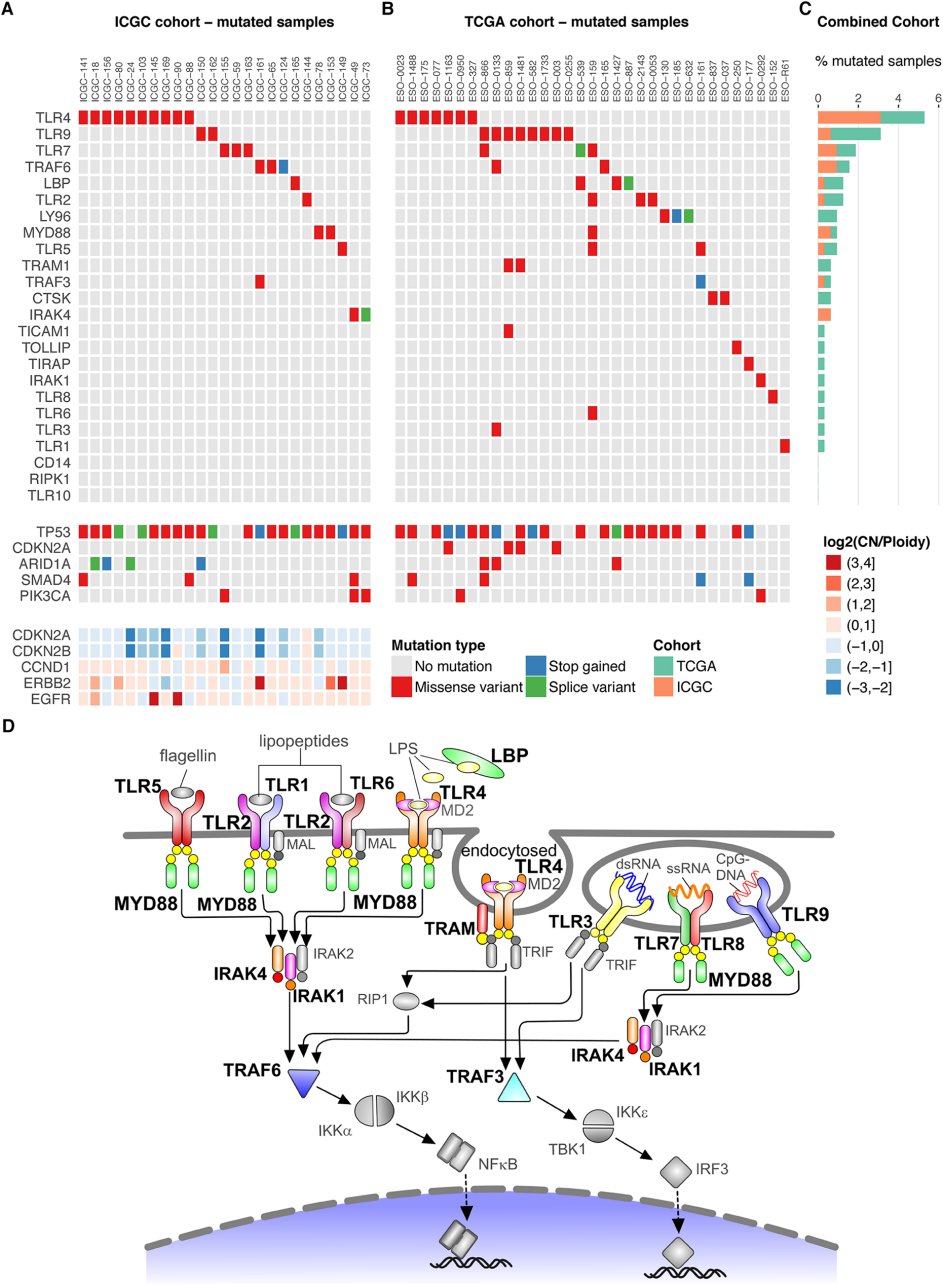
TLR pathway mutations in EAC. The EAC samples with TLR pathway mutations in the (A) ICGC cohort (n = 171 patients) and (B) TCGA cohort (n = 149 patients), with gene names along the vertical axis and sample names along the horizontal axis. The mutation status for known driver mutations in EAC (*TP53*, *CDKN2A*, *SMAD4*, *PIK3CA*) is presented for each sample with TLR pathway mutations in the ICGC and TCGA cohorts. Recurring oncogenic amplifications (*ERBB2*, *EGFR*, *CCND1*) and deletions (*CDKN2A/B*) that are known drivers in EAC are presented for the ICGC cohort. LY96 is the gene encoding MD2 protein. (C) Total TLR pathway mutation frequency from the combined ICGC and TCGA cohorts (n = 320), with gene names along the vertical axis and percent of mutated samples along the horizontal axis. (D) Schematic of the TLR signaling pathway summarizing the somatic mutations from the combined ICGC and TCGA cohorts, with mutated pathway components highlighted in color and large bold font. Grey pathway components have no TLR pathway mutations in either cohort.

To determine whether the TLR signaling pathway is dysregulated through somatic mutations in EAC, we interrogated the mutational profiles of 171 EAC tumors and matched germline controls that were sequenced as part of the International Cancer Genome Consortium (ICGC) esophageal study. Non-synonymous somatic mutations affected the Toll-like receptor signaling pathway in 25/171 (14.6%) of EAC samples. Missense mutations (and two splice variants) were identified in *TLR4* (5.8%), *TRAF6* (1.8%), *TLR7* (1.8%), *TLR9* (1.2%), *MYD88* (1.2%), *IRAK4* (1.2%), *LBP* (0.6%), *TRAF3* (0.6%), *TLR5* (0.6%), and *TLR2* (0.6%, Fig 1A, S1 Table). Applying the PathScan tool showed that genes in the TLR signaling pathway were significantly enriched for mutations (p = 8.7x10^-5^). Fig 1A presents the mutation and copy number status for known recurrent alterations in EAC in the context of TLR pathway mutated samples, and these events are compared with the remainder of the ICGC cohort in S1 Fig. As expected in EAC, most samples showed mutations in *TP53*, independent of whether or not they contain TLR pathway mutations. There was no significant enrichment of other known driver mutations in the TLR pathway mutated samples (S1 Fig). TLR pathway mutated tumors did not show significant differences in the numbers of total SNVs (Welch’s t-test, p = 0.134) or non-synonymous SNVs (p = 0.147) compared to wild-type tumors and did not show significant enrichment of any of the molecular subtypes recently defined on the basis of their dominant molecular signature (Fisher’s exact test, p = 0.57, S1 Fig) [20].

To investigate other potential sources of altered function of the TLR pathway genes, we investigated copy number and structural variants potentially affecting the TLR pathway in the ICGC cohort. Analysis of the copy number profiles of the TLR pathway genes indicated copy number gains for *LBP* and *CTSK*, while *TLR3*, *RIPK1* and *CD14* frequently showed fewer copies compared to the average ploidy (S2 Fig). Three samples showed homozygous deletions in TLR pathway genes: *MYD88* in ICGC-30, *IRAK1* in ICGC-24, and *TLR7*, *TLR8* and *IRAK1* in ICGC-10. Interestingly, several TLR pathway genes were affected by loss of heterozygosity. Loss of heterozygosity events overlapped with SNVs in *TLR4* in four samples (ICGC-24, ICGC-156, ICGC-18 and ICGC-141) and in *MYD88* in one sample (ICGC-78). Eleven out of 171 samples showed structural variants whose breakpoints overlap with TLR pathway genes or lead to a potential fusion with a TLR pathway gene (S2 Table). The following genes were affected: *TLR1*, *TLR3*, *TLR9*, *TLR10*, *RIPK1*, *TOLLIP*, *TRAF3*, *TRAF6*, *TRAM1* and *LBP*. Most structural variants were classified as duplications or deletions and there were three translocation and two inversion events. No recurrent events were detected.

### Validation of TLR pathway mutations in a second EAC cohort and other cancers

**Fig 2:**
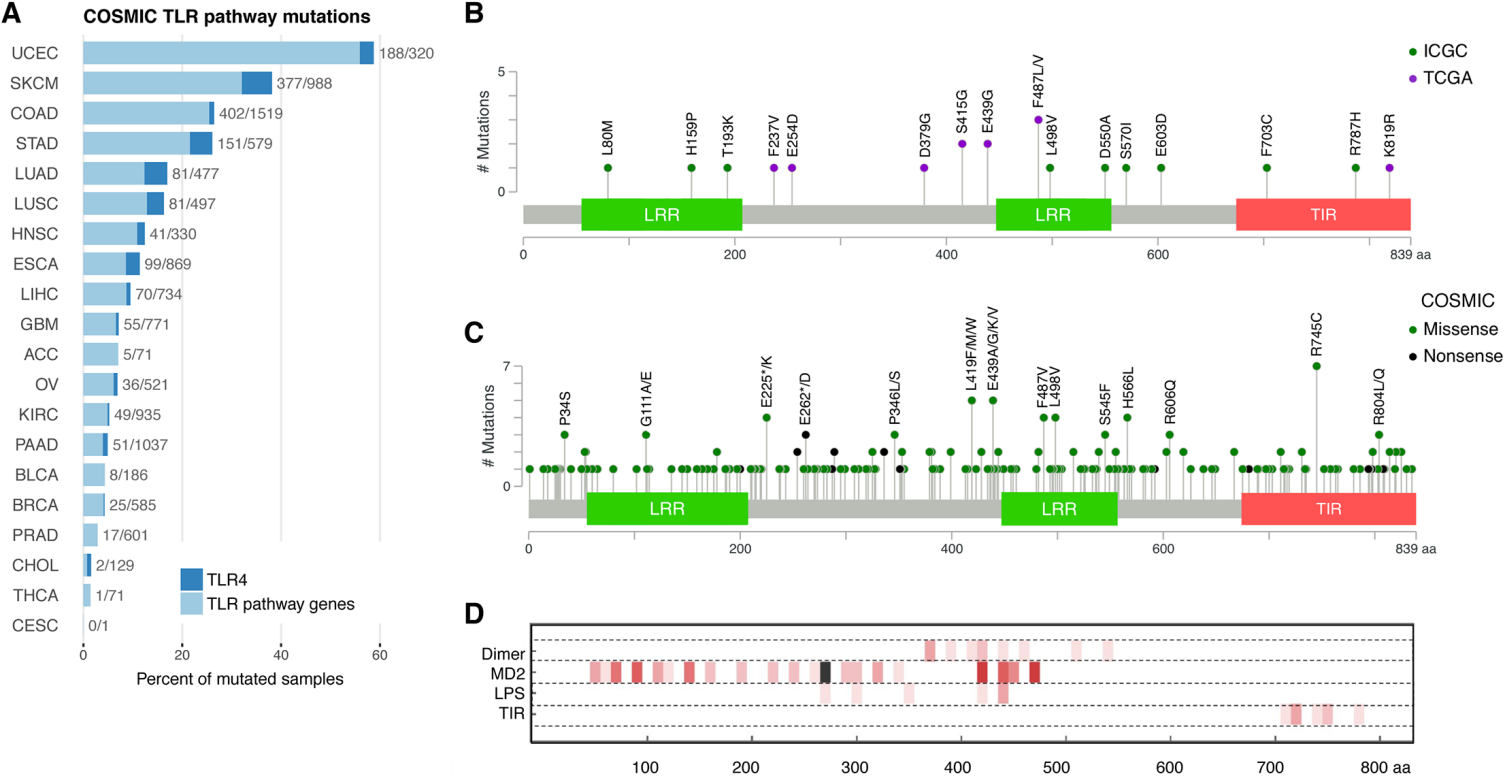
Distribution of TLR pathway mutations in EAC and other cancers. (A) TLR pathway mutations in different cancer types from the COSMIC database, with cancer types along the vertical axis and percent of mutated samples along the horizontal axis (abbreviations provided in S3 Table). *TLR4* mutations are indicated in dark blue. (B) Schematic of TLR4 protein with mutations from the ICGC (green circles) and TCGA (purple circles) EAC cohorts. Both EAC cohorts identified missense mutations at amino acid position E439 and F487. (C) Schematic of TLR4 protein with mutations from different cancer types in the COSMIC database. (D) Three lanes show dimer contacts (to other TLR4 chain), MD2, and LPS (PDB ID: 4G8A [22]). The fourth lane was calculated from a homology model of TLR4 TIR domain dimer (based on PDB ID: 2J67 [23]). Contacting TLR4 residues with atoms less than 0.5 Å apart at surfaces were determined using Chimera findclash function. Numbers of contacts are plotted in 10 residue sections of increasingly dark red tones (maximum 8 is black).

To ensure that the findings were generalizable we interrogated exome sequencing data from the Dulak et al. TCGA cohort [6] using our computational pipeline, and found a similar mutational spectrum with non-synonymous mutations in TLR pathway genes in 33/149 (22.1%, PathScan p = 0.05) of EAC samples (Fig 1B). Based on the combined data from both cohorts (Fig 1C and 1D), the most frequently mutated genes were *TLR4* (5.3%) and *TLR9* (3.1%), and these events appear to be mutually exclusive. We verified 11/11 *TLR4* mutations and 2/2 *TLR9* mutations from the ICGC cohort using PCR and Sanger sequencing (S3 Fig). We also examined the COSMIC database for 20 different cancer types (as defined in S3 Table), comprising a total of 10,318 samples, and found a high proportion of mutations in TLR pathway genes in endometrial carcinoma (188/320, 58.8%), cutaneous melanoma (377/988, 38.2%), colorectal adenocarcinoma (402/1519, 26.5%), stomach adenocarcinoma (151/579, 26.1%), lung adenocarcinoma (81/477, 17%), lung squamous cell carcinoma (81/497, 16.3%), head and neck squamous cell carcinoma (41/330, 12.4%), and esophageal carcinoma (combined adenocarcinoma and squamous cell carcinoma: 99/869, 11.4%, Fig 2A). The COSMIC database further showed a high proportion of *TLR4* non-synonymous mutations in cutaneous melanoma (60/988, 6.1%), lung adenocarcinoma (22/477, 4.6%), stomach adenocarcinoma (26/579, 4.5%) and lung squamous cell carcinoma (17/497, 3.4%, Fig 2A and S4 Table). Next we categorized the different cancer types into two groups based on tumors that arise in body sites associated with significant microbiomes, including the oral and gastrointestinal tract, skin, urogenital tract and respiratory tract [21], and those with rare exposure to microbes. There was an increase in the frequency of TLR pathway mutations in cancer types that are highly exposed to microbes (p = 0.019, Wilcoxon rank-sum test, S4 Fig). This trend was also present when looking only at the fraction of *TLR4* mutant tumors (p = 0.028).

### Clinical relevance of TLR pathway mutations

Next we investigated the clinical relevance of TLR pathway mutations through correlation with clinical outcome data. In the ICGC EAC cohort, patients with TLR pathway mutations (n = 25) tended to have more advanced disease with metastases (Fisher’s exact test, p = 0.036, Table 1). TLR mutant tumors originated more proximally at the level of the gastro-esophageal junction or above (Siewert Type 1–2 or esophageal, Fisher’s exact test, p = 0.012). There was a trend towards decreased survival in patients with TLR mutations in comparison to wild-type although this did not reach statistical significance, possibly due to the limited number of TLR mutant cases and length of follow-up (S5 Fig). This trend was also seen when comparing patients with TLR pathway mutations to wild-type patients in the EAC cohort from the TCGA database, although again the clinical data is limited.

**Table 1:** Clinicopathologic characteristics grouped by TLR pathway mutation status.

	TLR pathway mutation status			
	Mutation (n = 25)	No mutation (n = 146)	Overall (n = 171)	p-value
Mean Age (StDev)	65.7 (10.3)	66.6 (9.6)	66.5 (9.7)	0.679
Sex (% male)	88.0	85.6	86.0	1.000
Patient CTX (% treated)	75.0	77.2	76.9	0.797
Sample CTX (% treated)	24.0	37.9	37.4	0.180
Tumor location (%)				**0.012**
Esophageal	0.0	7.5	6.4	
GOJ Type 1	72.0	40.4	45.0	
GOJ Type 2	28.0	37.0	35.7	
GOJ Type 3	0.0	15.1	12.9	
Tumor Stage (%)				0.226
Stage 1	4.0	16.7	14.8	
Stage 2	24.0	13.9	15.4	
Stage 3	68.0	61.8	62.7	
Stage 4	4.0	7.6	7.1	
Node positive (%)	75.0	66.0	67.3	0.483
Metastasis (%)	26.3	8.6	10.9	**0.036**
Differentiation (%)				0.615
Well	0.0	2.1	1.8	
Moderate	54.2	43.4	44.9	
Poor	45.8	54.5	53.3	

Variables are compared for 171 patients who underwent whole genome sequencing in the esophageal ICGC study. Significant p-values are bolded. All p-values were calculated using Fisher’s exact test, except for age where the t-test was used. CTX = chemotherapy.

EAC frequently arises from the premalignant lesion Barrett’s esophagus through different degrees of dysplasia. To investigate the timing of TLR pathway mutations in disease pathogenesis, we examined samples from patients with Barrett’s esophagus adjacent to tumor (n = 24). The adjacent Barrett’s was non-dysplastic in 18/24 cases, contained low grade dysplasia in 2/24 cases and was indefinite for dysplasia in 2/24 cases. Only 2/24 Barrett’s samples showed mutations in the TLR pathway. A *TRAF6* mutation was present in tumor and adjacent Barrett’s, indicating that the mutation had occurred early in carcinogenesis. In another patient, a *TLR9* mutation was detected in Barrett’s but not the adjacent tumor. No other TLR pathway mutation was found in any of the Barrett’s adjacent to tumors, while three additional tumors had TLR pathway mutations.

### *In silico* analysis suggests *TLR4* mutations are potentially damaging

Since *TLR4* was frequently mutated in both EAC cohorts and other solid tumor types in the COSMIC database, we decided to characterize these mutations in greater detail. Both EAC cohorts identified missense mutations at amino acid position E439 (substitution to glycine) and F487 (substitution to leucine or valine, Fig 2B). The COSMIC database showed additional missense mutations at position E439 (two in stomach adenocarcinoma and one in cutaneous melanoma) and position F487 (two in stomach adenocarcinoma and one in esophageal carcinoma, Fig 2C). Seven tumors with *TLR4* mutations had paraffin-embedded tissue available to evaluate mutant TLR4 protein expression using immunohistochemistry for TLR4 monoclonal antibody. Similar to wild-type tumors, the *TLR4* mutant tumors showed combined membranous and cytoplasmic staining of TLR4 protein, with staining intensity ranging from weak to strongly positive (S6 Fig). None of the mutant tumors showed complete loss of *TLR4* expression, which was anticipated since the missense mutations did not cause truncation of the protein. We hypothesized that the mutations could have a functional effect on TLR4 signaling, and this was supported by computational modeling using a published crystal structure for dimerized human TLR4 ectodomain with associated MD2 co-receptor (LY96 gene product) with LPS bound (PDB ID 4G8A [22]) and a hypothetical structure for the dimerized TIR domain based on TLR10 (PDB ID 2J67 [23], S7 Fig). Two structurally significant mutations affected the TIR domain (F703C and R787H), which is involved in downstream signaling and interaction with the adaptor molecule MyD88 [14]. The E439G mutation is also critically located at the TLR4 dimerization interface and may disrupt hydrogen bonds in the binding site of LPS and MD2 (Fig 2D). Further, amino acid sequence alignment against seven non-human species showed that the positions of the TIR domain mutations (F703 and R787) are evolutionarily conserved, along with amino acids L80, L498 and S570, and E439 is semi-conserved (S8 Fig). Overall the combination of crystal structure modeling, sequence alignment, and SNP prediction algorithms (SIFT and Polyphen) suggested that six of the verified *TLR4* mutations could have a functional consequence: L80M, E439G, L498V, S570I, F703C and R787H (S5 Table).

### TLR4 mutations show decreased NF-κB activation upon stimulation *in vitro*

**Fig 3:**
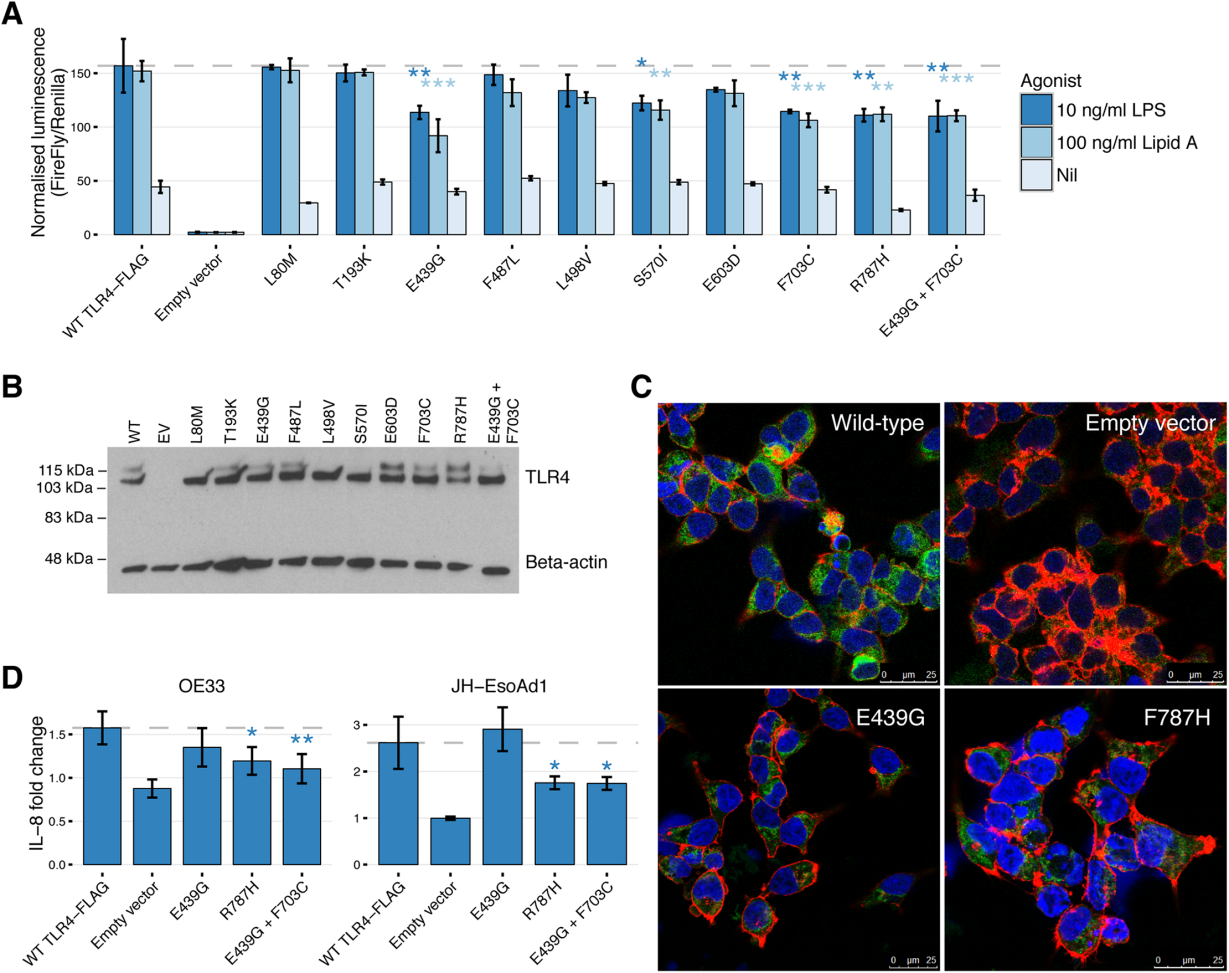
Functional analysis of *TLR4* mutations. (A) NF-κB response to TLR4 ligands in HEK293 cells transfected with *TLR4* mutants *versus* wild-type *TLR4* and stimulated with 100 ng/ml synthetic Lipid A and 10ng/ml LPS. The y-axis is normalized luminescence, calculated by dividing NF-κB firefly luciferase by the housekeeper renilla luciferase. Results are the average of three experiments with all conditions performed in triplicate. Error bars are standard deviation. *p<0.05, **p<0.01, ***p<0.001. (B) TLR4 protein expression 48 hours after transfection for NF-κB luciferase reporter assays. Representative Western blot shows protein expression for nine *TLR4* mutants, the double mutant (E439G + F703C), wild-type *TLR4* and empty vector. Fully glycosylated TLR4 is the upper band (120 kDa), and deglycosylated TLR4 is the lower band (110 kDa). (C) Immunofluorescence staining FLAG-AlexaFluor 488 antibody (green) in HEK293 cells transfected with wild-type TLR4-FLAG shows cytoplasmic distribution, in comparison to empty vector. Mutant E439G and R787H did not show any difference in localization of TLR4 protein. DAPI (blue) and Phalloidin (red) were used to visualize the cell nucleus and cytoskeleton, respectively. (D) Fold-change secretion of IL-8 for the EAC cell lines OE33 and JH-EsoAd1 following transfection of *TLR4* mutants E439G, R787H, E439G+F703C and wild type *TLR4*. Cells were stimulated with synthetic MPLA and LPS for 24 hours prior to ELISA. Data were normalized by dividing the concentration of IL-8 by the unstimulated (nil) value for each transfection condition. Data are an average of four independent experiments performed in duplicate. Error bars are standard deviation. *p<0.05, **p<0.01,

To test our functional predictions for the *TLR4* mutants, we performed site-directed mutagenesis and NF-κB luciferase reporter assays in HEK293 cells, a common model for measuring TLR signaling. The *TLR4* mutants were stimulated first using the weak agonist synthetic monophosphoryl Lipid A (MPLA). There was a significant decrease in ligand-dependent signaling for 7/9 of the *TLR4* mutations stimulated with synthetic MPLA (S9 Fig). A double mutation of E439G with F703C, representing a tumor with two *TLR4* mutations, showed a further decrease in TLR4 signaling compared to F703C (p = 0.0052) but not E439G (p = 0.099). Stimulation with stronger TLR4 agonists, synthetic Lipid A and lipopolysaccharide (LPS), showed a significant decrease in signaling for four single mutants (E439G, S570I, F703C and R787H) and the double mutation E439G + F703C (Fig 3A). Western blotting for recombinant FLAG-TLR4 confirmed adequate expression of the different *TLR4* mutants (Fig 3B). We also visualized recombinant TLR4-FLAG protein expression in HEK293 cells using confocal microscopy for mutants E439G and R787H, and there was no difference in expression or localization of TLR4-FLAG for either of the mutants in comparison to wild-type, suggesting that the decreased signaling was due to altered protein function rather than mis-folding and failure to reach the cell surface (Fig 3C).

Next, *TLR4* mutants E439G, R787H and E439G+F703C were transfected into EAC cell lines stimulated with LPS for 24 hours, and secretion of the NF-κB dependent cytokine IL-8 was measured. OE33 and JH-EsoAd1 cells were selected because of their low endogenous TLR4 mRNA expression and ability to secrete measurable amounts of IL-8 (S10 Fig). The fold change of IL-8 secretion was significantly lower for mutants R787H and E439G+F703C in comparison to wild-type *TLR4* (Fig 3D). In contrast to HEK293 cells, no significant decrease in TLR4 signaling was observed for mutant E439G stimulated with LPS in the EAC cell lines, suggesting that the strong agonist LPS was still able to trigger TLR signaling despite mutation of the ligand binding site.

### Upregulation of NLRP6 expression in EAC tumors with TLR pathway mutations

**Fig 4:**
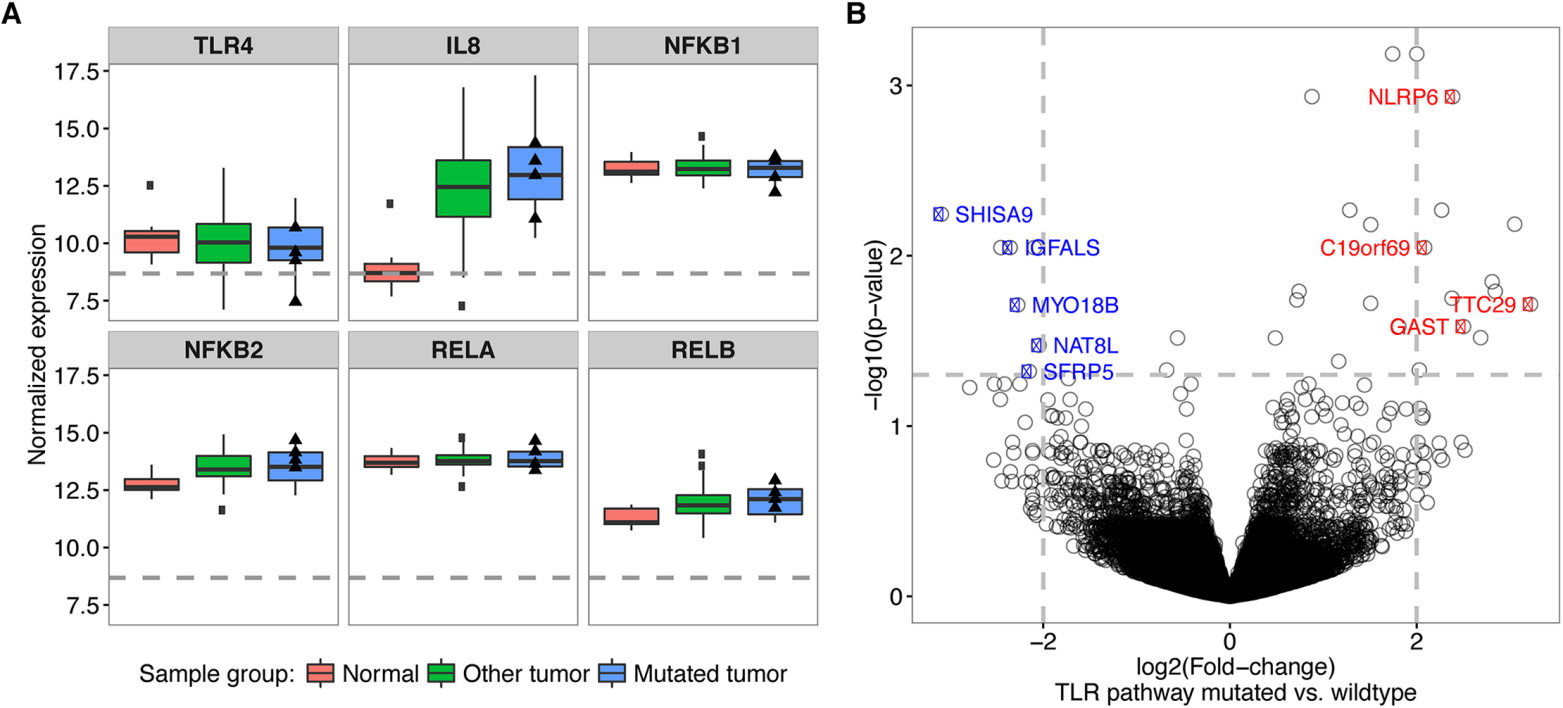
Gene expression in EAC tumors with TLR pathway mutations. (A) RNA-Seq data for TLR4, IL-8 and NF-κB genes in the TCGA cohort. The 89 EAC samples were split into TLR pathway mutated (n = 17) and wild-type samples (n = 72) using the matched mutation data. The y-axis shows the gene expression data normalized by the library size with the median by sample group indicated as a solid black line within each box. The lower edge and the upper edge of the boxes represent the first and the third quartile of the expression value distribution, respectively. The lower whisker represents the lowest value within 1.5-times the inter-quartile range from the lower edge of the box and the upper whisker the highest value accordingly. Dots indicate outliers to these ranges and triangles indicate the expression values of samples with a TLR4 mutation. The horizontal dashed grey line indicates the average expression value of all genes. (B) Vulcano plot showing the–log10-transformed, adjusted p-values from the differential expression test on the y-axis and the estimated log2-transformed fold-change between TLR mutated vs. wild-type samples on the x-axis. The grey dashed lines show the fold-change and p-value cutoffs applied to detect significantly differentially expressed genes. The colored and labeled points indicate down-regulated (blue) and upregulated (red) genes in TLR pathway mutated samples as compared to the wild-type and to the normal samples.

Our experiments in cell lines suggest that *TLR4* mutations impair TLR signaling and NF-κB activation in HEK293 cells and IL-8 secretion by EAC cell lines. We next tested whether there was any effect on gene expression in patient data using RNA-Seq data available for the TCGA cohort. Out of 89 samples with RNA-Seq data available, 17 samples had TLR pathway mutations and four had *TLR4* mutations. Our analysis using DESeq2 [24] shows that expression of IL-8, NFKB2 and RELB was significantly elevated in the tumors compared to normal samples (n = 10), but no significant difference was observed when comparing tumors with mutations in the TLR pathway and wild-type tumors, possibly related to the small sample size (Fig 4A). Similarly, there was no significant difference in gene expression between *TLR4* mutant and wild-type tumors. We next searched for alternative genes whose expression could be affected by TLR mutations *in vivo*. Tumors with TLR pathway mutations showed significant upregulation of NLRP6, GAST, TTC29 and C19orf69, and down-regulation of SFRP5, MYO18B, NAT8L, SHISA9 and IGFALS (Fig 4B). We validated our findings using RNA-Seq data from 23 independent tumors of which 2 were mutated in the TLR pathway, and significant upregulation was found only for NLRP6 (p = 0.004). NLRP6 is involved in pathogen recognition through the inflammasome pathway and has overlap in function with the TLR pathway. Additionally, quantitative RT-PCR was performed in 22 tumor samples with available RNA (11 of which contained TLR pathway mutations, including 5 *TLR4* mutations). The results showed a similar trend, with upregulation of NLRP6 in TLR pathway mutated samples in comparison to wild-type tumors; however, this did not reach significance likely due to the small sample size (p = 0.172, S11 Fig). Of the samples with *TLR4* mutations, R787H (relative expression 6.8) and L80M (relative expression 3.7) showed upregulation of NLRP6 compared to mean expression in wild-type tumors (relative expression 0.87, S6 Table).

## Discussion

Our results suggest that somatic mutations may collectively target the TLR signaling pathway in EAC (25% of cases) and other solid tumor types. TLR mutant cases were associated with more proximal tumors and metastatic disease, indicating possible clinical significance of these mutations. *TLR4* was the most frequently mutated TLR gene in 5% of cases in the combined EAC cohorts. Only two TLR pathway mutations (*TLR9* and *TRAF6*) were detected in the adjacent Barrett’s samples in the ICGC cohort. *TLR4* mutations have been previously reported in high grade dysplasia [5], which implies that this mutation may be acquired later during disease progression. Although *TLR4* is mutated in less than ten percent of EAC cases, it is one of the top twenty most frequently mutated genes in both the ICGC and TCGA cohorts. The most frequently mutated gene in this disease is *TP53*, which is mutated in approximately 70% of tumors [6]. A limited number of candidate driver genes have been identified that are mutated in greater than ten percent of EAC tumors, including *CDKN2A*, *SMAD4*, *ARID1A*, *MYO18B* and *DOCK2* [5,6].

In addition to EAC, TLR pathway mutations were frequently observed in solid tumors arising in body sites exposed to microbiota [21] including the oral and gastrointestinal tract (colorectal adenocarcinoma, stomach adenocarcinoma and head and neck squamous cell carcinoma), skin (melanoma), urogenital tract (endometrial carcinoma) and respiratory tract (lung adenocarcinoma and squamous cell carcinoma). Regrettably, it was not feasible to analyze our whole genome sequencing data using pathogen discovery software such as PathSeq [25], because the concentration of microbial DNA is so low relative to human DNA in samples derived from esophageal tumor tissue and the results were unreliable with high levels of contaminants. This is a major limitation of analyzing low microbial biomass tissue samples with a WGS approach. Our recent work examining the microbiome in esophageal tumors and Barrett’s tissue samples used 16S rRNA gene amplicon sequencing and a microbial extraction protocol with rigorous reagent controls to maximize the microbial DNA yield and account for contaminants [13]. However, only one of the tumors from that study had a *TLR4* mutation so it was not possible to further correlate the findings with our current study, and additional research is needed to investigate the link between TLR mutations and the microbiome in EAC.

Seven *TLR4* mutants in our study showed decreased signaling in response to weak agonists, and four were hypo-responsive to strong agonists. This suggests that the mutations differentially affected signaling with weak *versus* strong agonists, which may be relevant to the different microbial antigens present in the tumor environment. The model system was also a contributing factor; for instance E439G was hyporesponsive to LPS in HEK293 cells but not EAC cell lines. In EAC cell lines, addition of the second mutation F703C was required to significantly reduce TLR4 signaling activity. Further, loss of heterozygosity events overlapped with four *TLR4* mutations, R787H, E603D, T193K, and L498V. Of these, R787H showed decreased signaling in response to strong and weak agonists, L498V showed decreased signaling in response to weak agonist only, and E603D and T193K showed no significant change. It is conceivable that loss of heterozygosity could potentially compound the effect of *TLR4* missense mutations and further decrease signaling. A possible mechanism is that defective TLR4 signaling may negatively impact epithelial cell repair, which is in part dependent on TLR4 stimulation, and potentially enable microbes to breach the epithelial barrier in the tumor microenvironment. Hold et al. found that the single nucleotide polymorphism c.896A>G (p.D299G) was associated with an increased risk of gastric cancer in two patient populations [19], and cells with this mutation have been shown to be hypo-responsive to lipopolysaccharide [26,27]. However, Hold et al. found no significant increased risk for EAC or esophageal squamous cell carcinoma with this germline polymorphism, and the D299G polymorphism was not identified in either of the ICGC or TCGA cohorts.

The relationship between TLR4 signaling and tumorigenesis is complex and involves both innate and adaptive immunity, with evidence showing that TLR4 signaling can enhance or suppress cancer development, depending on the model system. For example, in the setting of chemically induced colitis *TLR4* deficient mice were protected from colon carcinogenesis [28], while villin-*TLR4* mice overexpressing TLR4 in the intestinal epithelium were highly susceptible to cancer when treated with dextran sodium sulphate and azoxymethane [29]. Conversely, overexpressing TLR4 using the CD4-*TLR4* transgene in the intestinal epithelium of APC^Min/+^ mice that are genetically susceptible to colon carcinoma reduced tumorigenesis by increasing apoptosis [30]. Additionally, analysis of TCGA expression data suggested that NLRP6 is upregulated in TLR mutant tumors, which may reflect cross-talk between the TLR pathway and NOD-like receptor signaling pathway. NLRP6 regulates inflammasome-dependent innate immune signaling and has been shown to inhibit TLR2 and TLR4-dependent activation of NF-κB and MAP-kinase signaling pathways [31]. Further understanding of how altered TLR signaling may contribute to the inflammatory tumor microenvironment in Barrett’s carcinogenesis could be helpful in cancer prevention strategies.

## Materials and methods

### Ethics statement

Ethical approval was obtained from the National Research Ethics Services Cambridgeshire Research Ethics Committee on behalf of all hospital centers in the OCCAMS/ICGC trial (REC 07/H0305/52 and 10/H0305/51). Written informed consent was obtained from all subjects prior to the collection of samples and recording clinical information.

### Human samples

EAC tissue samples with matched germline controls (n = 171 patients) were collected from 11 UK hospitals participating in Oesophageal Cancer Clinical and Molecular Stratification (OCCAMS) for the International Cancer Genome Consortium (ICGC). Ethical approval was obtained from the National Research Ethics Services Cambridgeshire Research Ethics Committee on behalf of all hospital centers in the OCCAMS/ICGC trial (REC 07/H0305/52 and 10/H0305/51). Written informed consent was obtained from all subjects prior to the collection of samples and recording clinical information. All tissue samples were flash frozen in liquid nitrogen and stored at -80°C. The tissue histology was reviewed by an expert gastrointestinal pathologist to ensure that tumor cellularity was greater than seventy percent in samples selected for sequencing.

### Whole genome sequencing

DNA was extracted using the QIAGEN AllPrep kit and quantified using the Qubit Fluorometer. Whole genome sequencing was performed on the Illumina HiSeq platform in San Francisco, CA, USA. 100 bp paired-end sequencing was performed to an average depth of 50-fold for tumors and 30-fold for controls, achieving a Phred quality score of at least 30 for 80% of mapping nucleotide bases. WGS sequencing data has been deposited in the European Genome-phenome Archive under accession number EGAD00001001960: https://www.ebi.ac.uk/ega/search/site/EGAD00001001960.

### Variant calling

Single-nucleotide variant calling was performed as described previously [20]. Briefly, reads were mapped to the human reference genome (GRCh37) using BWA 0.5.9 [32]. Mutations and Indels were called using Strelka 1.0.13 [33]. The resulting SNVs were filtered using a custom set of filters [20] and then annotated using the Variant Effect Predictor (VEP release 75) tool [34]. As described in Secrier et al. [20], for structural variant calling the reads were mapped to the GRCh37 reference genome, and Manta [35] was used to identify putative breakpoint junctions using discordant read pairs and split reads. Breakpoints and potential run-through events were annotated using the Ensembl GRCh37, version 75 gene annotation.

### Copy number calling and loss of heterozygosity

Copy number calling was performed as described by Secrier et al. [20] In short, absolute and minor copy number of genomic loci was estimated using ASCAT-NGS v2.1 [36]. Read counts at germline heterozygous positions were estimated by GATK 3.2–2 [37]. ASCAT estimates for tumor purity and average ploidy for each sample were used. Loss of heterozygosity and homozygous deletions were defined for loci with an estimated minor copy number of 0 and an estimated total copy number of 0, respectively. For each gene the highest copy number change is shown. The copy number alterations for genes were classified into gains, losses, amplifications and deletions based on the relative copy number rCN = log2(CN/ploidy), where CN is the copy number of the gene and ploidy is the average ploidy as estimated by ASCAT. The cut-offs for classification are: deletion–rCN< = -2, loss—rCN< = -1, no change—-1>rCN<1, gain—rCN> = 1, amplification—rCN> = 2.

### TLR pathway analysis

We defined a list of genes that are specific to the TLR pathway based on the KEGG Toll-like receptor signaling pathway. PathScan [7] was used to assess whether mutations affecting the TLR pathway were significantly enriched in the ICGC cohort or the Dulak et al. TCGA cohort [6]. We used a Docker image (https://hub.docker.com/r/beifang/music/) of the Genome MuSiC 0.4 suite [38] to run PathScan. The overall background mutation rate was calculated on non-synonymous mutations using the human genome GRCh37 and all human Ensembl genes as a reference. The mutational consequences of each SNV predicted by VEP were converted to MAF format using the conversion scheme of vcf2maf (https://github.com/mskcc/vcf2maf/blob/master/vcf2maf.pl). The COSMIC database v78 (http://cancer.sanger.ac.uk/cosmic) was downloaded and interrogated for *TLR4* and TLR pathway mutations. We only used mutations derived from high-throughput studies. Further, we selected only missense and nonsense substitutions, as well as frameshift insertions or deletions. Mutations were counted by cancer type as defined by the filters for tissue type and histology described in S3 Table.

### Sample classification based on mutational signatures

Samples were grouped into molecular subtypes as defined by Secrier et al. [20] The mutational context of each mutation was defined using the UCSC hg19 reference genome and the R package SomaticSignatures [39]. The estimated mutational signatures from Secrier et al. were used to fit the exposures by non-negative least squares. The three molecular subgroups were defined by consensus clustering on the estimated exposures.

### Structural modeling of TLR4 mutations

The ICGC TLR4 mutations were modeled using mutation functions in COOT (Crystallographic Object-Oriented Toolkit) [40] applied to the crystal structure for dimerized TLR4 ectodomain (PDB ID: 4G8A [22]) bound to MD2 and lipopolysaccharide (LPS) and a hypothetical structure for the Toll/interleukin-1 receptor (TIR) domain based on the TLR10 dimer (PDB ID: 2J67 [23]). The starting ectodomain PDB structure 4G8A was the common human variant (D299G and T399I), which was initially mutated back to D299 T399 before modeling the observed ICGC mutations. The TLR10 TIR domain template had a 35% identity (61% similarity with BLOSUM50 matrix) over the 143 residues of the TLR4 TIR domain. TLR4 ectodomain model energy minimization, was with FoldX 3.0 [41], and TIR dimer contacts were optimized using PyRosetta [42]. Contacting TLR4 residues less than 0.5 Å apart at surfaces were determined using UCSF Chimera and sorted by residue range in Mathematica(TM) to show dimer contacts (to other TLR4 chain), interactions with the ligand lipopolysaccharide (LPS) and adaptor MD2. Amino acid sequence alignment for human TLR4 was performed using Uniprot (http://www.uniprot.org/align/) and Espript (http://espript.ibcp.fr/ESPript/cgi-bin/ESPript.cgi) against seven non-human species.

### PCR verification of *TLR4* mutations

Primers were designed to amplify an area approximately 200 bp in size surrounding *TLR4* mutations using Primer3 (S7 Table). PCR amplification was performed with Q5 Hot Start High Fidelity Taq and the following conditions: denaturation at 98°C for 30 s, 35 cycles of 98°C for 10 s, 55–60°C for 10 s and 72°C for 10 s, followed by a final extension at 72°C for two minutes. The PCR product was sent for Sanger sequencing (Source BioScience, Cambridge, UK).

### Vectors and cell lines

The plasmids pNF-κB-luc (firefly luciferase), pEFIRES-*MD2*, phRG-TK (renilla luciferase), pcDNA3-*CD14*, and a pCMV vector containing *TLR4* cDNA with an N-terminal FLAG tag (pCMV-*TLR4*-FLAG) were provided by N. Gay [43]. HEK293 cells were cultured in Dulbecco’s Modified Eagle’s Medium (DMEM: Gibco, Life Technologies Ltd.) supplemented with 4.5 g/l glucose, 10% foetal bovine serum (FBS: HyClone Laboratories Inc.), penicillin (50 U/ml) and streptomycin (50 μg/ml) at 37°C, 5% CO_2_. The EAC lines OE33 (European Collection of Cell Cultures (ECACC)), SKGT4 (ECACC), OE19 (Sigma-Aldrich), OACP4C and OACM5.1 (both provided by W. Dinjens [44]) were cultured in RPMI-1640 medium (Sigma-Aldrich) supplemented with 10% FBS. JH-EsoAd1 cells were obtained from H. Alvarez [45] and grown in Minimal Essential Medium (MEM: Gibco) supplemented with 10% FBS. HEK293-hTLR4-MD2-CD14 cells (InvivoGen) were cultured in DMEM with 4.5 g/l glucose, 10% FBS, 50 U/ml penicillin, 50 μg/ml streptomycin and 100 μg/ml Normocin (InvivoGen). Selective antibiotic pressure was maintained with 10 μg/ml Blasticidin (InvivoGen) for hTLR4 and 50 μg/ml Hygrogold (InvivoGen) for MD2 and CD14 plasmids. FLO-1 cells (from D. Beer [46]) were cultured DMEM with 4.5 g/l glucose and 10% FBS.

### Site-directed mutagenesis

Site-directed mutagenesis was performed using the Stratagene QuikChange II Kit and *PfuUltra* HF polymerase (Agilent Technologies, Inc.). Primers were designed using PrimerX with the mutation site at the center region (S7 Table). Mutagenesis was confirmed by Sanger sequencing the entire *TLR4* cDNA. NOT1 and BamH1 (New England Biolabs) were used for cloning, and ligation was performed with T4 DNA Ligase (New England BioLabs) using an insert:vector ratio of 3:1.

### NF-κB reporter gene assays

HEK293 cells were seeded at 20,000 cells per well in 96-well plates and transfected with 0.2 μl jetPEI (Polyplus Transfection) per well and the expression plasmids (S8 Table), in triplicate. Forty-eight hours later, the cells were stimulated for six hours with 10 ng/ml LPS from *E*. *coli* O127:B8 (Sigma-Aldrich, L3129), 100 ng/ml synthetic Lipid A (PeptaNova, 24005-s) or 5 ng/μl synthetic monophosphoryl Lipid A (Enzo Life Sciences, ALX-581-205-C100) diluted in filtered DMEM with 10% FBS. Following lysis with passive lysis buffer (Promega) the luminescence of firefly and renilla luciferase was quantified with luciferin and coelenterazine reagents, respectively, using a FLUOstar OPTIMA microplate reader (BMG LABTECH).

### Quantitative reverse transcription PCR (qRT-PCR)

Total RNA was extracted using the AllPrep mini kit (QIAGEN), and 1 μg of RNA was transcribed into cDNA using the Quantitect reverse transcription kit (QIAGEN) and diluted 1:5 in nuclease free water. Two μl of cDNA were amplified in a 10 μl reaction volume containing 500 nM of each primer and 5 μl SYBR Green I Master (Roche). The primers for TLR4 spanned across two exons and the sequences were 5’CCGACAACCTCCCCTTCTCA (forward primer) and 5’GGCTCTGATATGCCCCATCTTC (reverse primer). The sequences for NLRP6 primers were 5’cctggtgggtatgcttcgg (forward primer) and 5’ctcctgtagtgactgctcgc (reverse primer). The LC480 LightCycler 480 II (Roche) template for SYBR Green 384 well plates was used with 45 amplification cycles of 10 s each for 95°C, 60°C, 72°C. All reactions were performed in triplicate. The cycle threshold (C_T_) results were normalized by subtracting the average of three housekeeping genes: ribosomal protein S18 (RPS18: forward 5'ATCCCTGAAAAGTTCCAGCA, reverse 5'CCCTCTTGGTGAGGTCAATG), beta-actin (ACTB: forward 5’GGCACCCAGCACAATGAAGA, reverse 5’ AAGCATTTGCGGTGGACGAT) and glyceraldehyde 3-phosphate dehydrogenase (GAPDH: forward 5’GTCTCCTCTGACTTCAACAGCG, reverse 5’ACCACCCTGTTGCTGTAGCCAA). The delta-delta C_T_ method [47] was used to compare relative mRNA expression between cell lines.

### Western blot

Protein lysate was separated using SDS-PAGE and blotted onto an Immobilon-P membrane (Fisher Scientific UK Ltd.). The membrane was probed with monoclonal rabbit antibodies for FLAG (1:1000, Sigma-Aldrich, F2555) and β-actin (1:10,000, New England Biolabs, 4970L), and incubated with peroxidase conjugated Pierce goat anti-rabbit IgG, H+L (1:2500, Fisher Scientific Ltd., 31462) secondary antibody. Bands were visualized using Amersham ECL Prime detection reagents (GE Healthcare UK Ltd.) developed on Kodak Biomax XAR film. Size of glycosylated TLR4 (120 kDa) and deglycosylated TLR4 (110 kDa) is based on da Silva Correia et al. [48].

### Enzyme-linked immunosorbent assay

Cells were seeded at 20,000 cells per well in 96-well plates and transfected with 0.25 μl Lipofectamine 2000 (Polyplus Transfection) per well and the expression plasmids (S9 Table). Twenty-four hours after transfection, cells were stimulated with 10 ng/μl monophosphoryl Lipid A or 40 ng/ml LPS diluted in culture medium. Secretion of IL-8 was measured using human Quantikine ELISA kits (R&D Systems Europe Ltd.) in duplicate.

### Confocal microscopy

Cells were seeded at 40,000 per well on coverslips in 24-well plates and transfected with 1 μl jetPEI per well and the appropriate transfection vectors (S10 Table), in triplicate. Forty-eight hours after transfection the cells were washed with PBS and fixed with 4% paraformaldehyde in PBS for 30 minutes at room temperature. The cells were washed twice and permeabilized with 0.2% Triton X-100 in PBS for 20 minutes at room temperature. The cells were washed three times with PBS and blocked with 2% bovine serum albumin and 5% chick serum in PBS for one hour at room temperature. The cells were stained with Alexa Fluor 488 conjugated DYKDDDDK (FLAG) Tag antibody (Cell Signaling Technology, 5407) diluted 1:200 in blocking buffer overnight at 4°C protected from light. The cells were washed three times with 0.1% TWEEN 20 in PBS (PBS-T) and incubated with Atto 594 Phalloidin (Sigma-Aldrich, 51927) diluted 1:100 in PBS for 20 minutes at room temperature protected from light. The cells were washed with PBS-T and stained with DAPI in PBS (1:5000) for 15 minutes at room temperature protected from light. The coverslips were inversely mounted on slides with hard-dry DPX mountant (Leica Biosystems). A Leica TCS SP5 II confocal microscope (Leica Microsystems) was used to image the cells.

### RNA-Seq analysis

RNA-Seq read counts per gene and variant calls for esophageal cancer were downloaded from the TCGA data portal (https://gdc.cancer.gov/). Only esophageal adenocarcinoma samples that had matched genotype data available and normal control samples were selected for the analysis. ICGC RNA-Seq bam files in Secrier et al. [20] for samples matching our cohort were also used. Reads overlapping RefSeq genes were counted in R using the GenomeAlignment package [49]. Entries with duplicated gene names were summarized to the median read count per sample. Only genes having at least one read count in any of the samples were retained. Differential expression analysis between TLR pathway mutated vs. wild-type, TLR pathway *vs*. control and wild-type *vs*. control samples was performed using DESeq2 [24].

### Statistical analysis

The analysis for luciferase reporter assays was performed using the ANOVA test and the Tukey post-test in Graphpad Prism 6. Within Graphpad, the nonparametric Kruskal-Wallis test and Dunns post-test was used to compare means for ELISA assays. Kaplan-Meier curves for survival analysis were generated using the log-rank test. The Wilcoxon rank-sum test was used to compare the frequency of TLR mutant samples for microbiome exposed *versus* rarely exposed tumors in the COSMIC database. A significant p-value was defined as less than 0.05.

## Supporting information

S1 Table Summary of TLR signaling pathway mutations in EAC samples from the ICGC cohort. *Median variant allele frequency of SNVs in regions with same copy number profile. **Average number of mutated alleles per cell (methods described by Landau et al. [50])
(XLSX)

S2 Table Structural variants involving TLR pathway genes. (XLS)

S3 Table Different cancer types from the COSMIC database with search terms and microbiota exposure. *Tumor types arising in body sites with significant microbiomes, including the oral and gastrointestinal tract, skin, urogenital tract and respiratory tract [21].
(XLSX)

S4 Table Number of samples by cancer type with a mutation in TLR pathway genes in the COSMIC database. Entries larger than 5 are highlighted in bold.
(XLSX)

S5 Table Predicted functional effects of *TLR4* mutations that were verified by Sanger sequencing n the ICGC cohort. For SIFT a low score (0) suggests a deleterious consequence, while a higher score (1) indicates the mutation is well tolerated. For PolyPhen a low score (0) indicates a benign mutation, while a high score (1) indicates a damaging mutation.
(XLSX)

S6 Table Relative expression of NLRP6 mRNA in TLR4 mutant tumor samples. (XLSX)

S7 Table Primer sequences. (XLSX)

S8 Table Amounts of plasmids transfected into HEK293 cells for NF-κB luciferase reporter gene assays in 96-well plates. (XLSX)

S9 Table Amounts of plasmids transfected into esophageal adenocarcinoma cell lines for ELISA assays in 96-well plates. (XLSX)

S10 Table Amounts of plasmids transfected into HEK293 cells in 24-well plates for confocal microscopy. (XLSX)

S1 Fig Comparison of genetic alternations for TLR pathway mutated tumors and wild-type tumors. (A) Copy number alterations in known driver genes in EAC. (B) Mutations in known driver genes in EAC. (C) Overall number of non-synonymous SNVs. (D) Molecular subtypes defined by Secrier et al. [20]
(TIF)

S2 Fig The predicted total copy number of a gene analyzed with respect to the estimated average ploidy of the sample. The copy number of the gene relative to the average ploidy estimated by ASCAT is shown. LOH = loss of heterozygosity. LY96 is the gene encoding MD2 protein.
(TIF)

S3 Fig Verification of eleven *TLR4* mutations and two *TLR9* mutations from the ICGC cohort using PCR and Sanger sequencing. Tracings are shown for tumor and matched normal germline. Nucleotide change is in the format reference base > mutant base.
(TIF)

S4 Fig Comparison of the frequency of TLR pathway mutations and TLR4 mutations in tumors that occur in body sites exposed to microbiota *versus* rarely exposed to microbiota. There is an increase in the frequency of mutated samples in cancer types that are highly exposed to microbes for TLR pathway mutant samples (p = 0.019, Wilcoxon rank-sum test) and TLR4 mutant samples (p = 0.028).
(TIF)

S5 Fig Kaplan-Meier survival analysis for EAC patients with TLR pathway mutations in the ICGC and TCGA cohorts. (TIF)

S6 Fig Immunostaining with TLR4 monoclonal antibody on seven tumors with *TLR4* mutations and two representative wild-type tumors in the ICGC cohort. The slides were scanned at 20X magnification and imaged at 20% digital zoom (equivalent 20X). The high magnification insets were imaged at 50X.
(TIF)

S7 Fig Structural modeling of nine TLR4 mutations from the ICGC cohort. The crystal structure of dimerised human TLR4 ectodomain (colored blue to orange, PDB ID: 4G8A [22]) has been linked by transmembrane alpha helices (grey) to a hypothetical TIR domain (colored orange to red) dimer based on the known crystal structure for TLR10 (PDB ID: 2J67 [23]). MD2 is colored purple and the atoms of the bound LPS are pink. Oxygen atoms are colored pink, and nitrogen atoms are blue. Black and grey spheres indicate the *TLR4* mutations on opposite sides of the TLR4 molecule. In detailed views (i) to (ix) the mutated sidechain atoms are distinguished by color (unmutated sidechain carbon atoms are in domain colors; mutated sidechain atoms are in standard atom colors, carbon gray).
(TIF)

S8 Fig Amino acid sequence alignment for human TLR4. Global sequence alignment to seven non-human species (mouse, gorilla, orangutan, rat, bovine, equine and porcine) was performed using Uniprot (http://www.uniprot.org/align/) and Espript (http://espript.ibcp.fr/ESPript/cgi-bin/ESPript.cgi). Evolutionarily conserved amino acids are highlighted in orange with red text, semi-conserved amino acids have red text only, and non-conserved amino acids have black text only. The secondary structure annotation is from the model in S7 Fig (PDB ID: 4G8A [22] and PDB ID: 2J67 [23]).
(TIF)

S9 Fig NF-κB luciferase reporter gene assay for *TLR4* mutants *versus* wild-type *TLR4*. HEK293 cells were stimulated with 5 ng/μl synthetic monophosphoryl Lipid A (MPLA). The y-axis is normalized luminescence, calculated by dividing NF-κB firefly luciferase by the housekeeper renilla luciferase. Results are the average of three experiments with all conditions performed in triplicate. Error bars are standard deviation. *p<0.05, **p<0.01, ***p<0.001.
(TIF)

S10 Fig Endogenous TLR4 expression and IL-8 secretion in esophageal cell lines. (A) Endogenous levels of TLR4 mRNA expression quantified using qRT-PCR in seven EAC lines, positive control HEK293-hTLR4-MD2-CD14 cells and negative control HEK293 cells. Relative mRNA expression is calculated as fold change relative to OE19 (defined as 1). Zero indicates an undetectable level of TLR4 (cycle threshold value greater than 35). Data are averages of three independent experiments. (B) ELISA for endogenous secretion of IL-8 in seven EAC lines, in the absence of any stimulation. Data are averages of two independent experiments, each performed in technical duplicate. Error bars are standard deviation.
(TIF)

S11 Fig NLRP6 mRNA expression quantified using qRT-PCR in RNA extracted from TLR pathway mutant and in comparison to wild-type tumors. Data are an average of three technical replicates.
(TIF)
