# Supplementary figures and images for: Impact of mutations in Toll-like receptor pathway genes on esophageal carcinogenesis
Source: PLoS Genet. 2017 May 22;13(5):e1006808. doi: 10.1371/journal.pgen.1006808 (PMC5460900; doi:10.1371/journal.pgen.1006808)

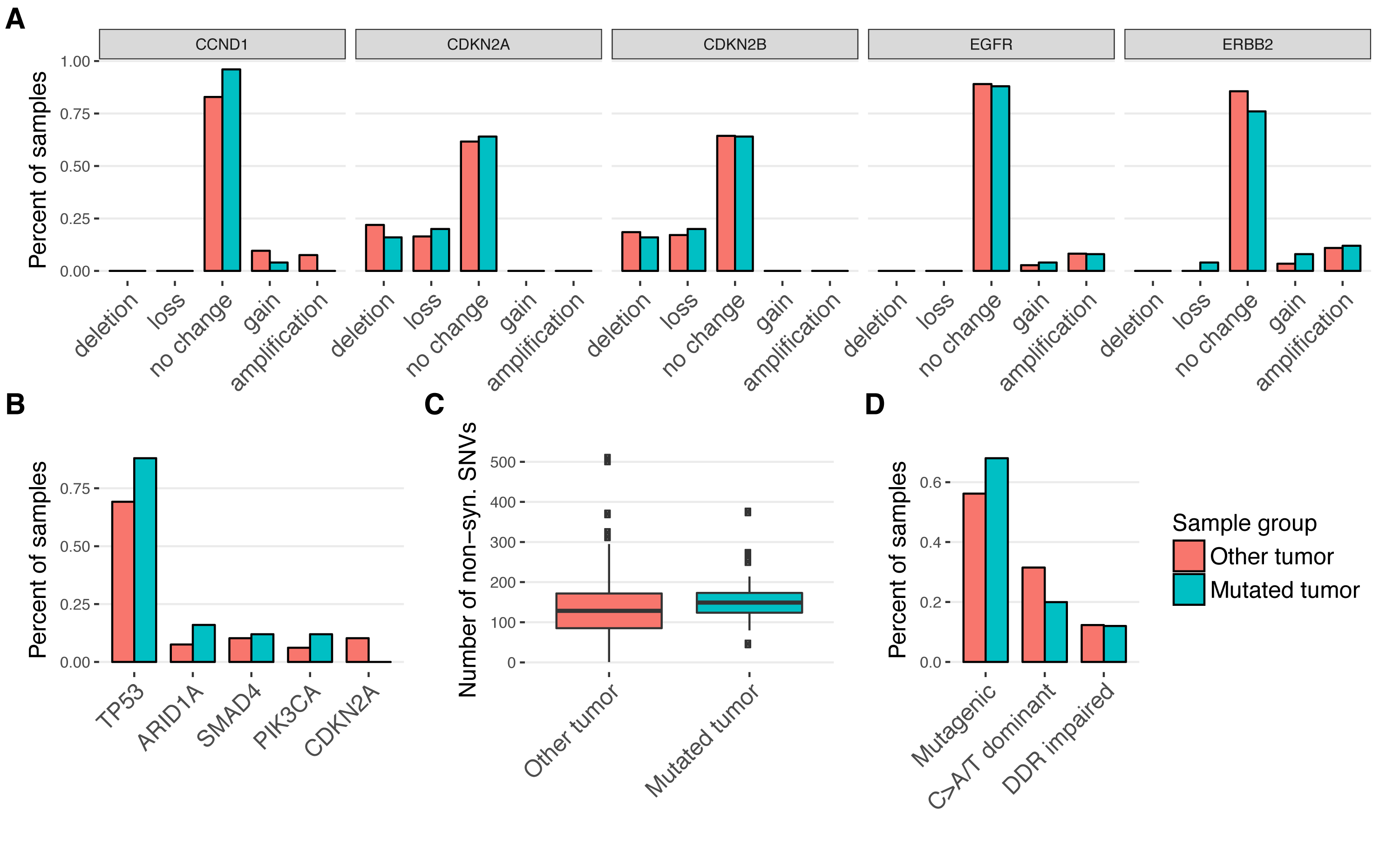

Supplement: S1 Fig — (A) Copy number alterations in known driver genes in EAC. (B) Mutations in known driver genes in EAC. (C) Overall number of non-synonymous SNVs. (D) Molecular subtypes defined by Secrier et al. [20] (TIF) [file pgen.1006808.s011.tif]

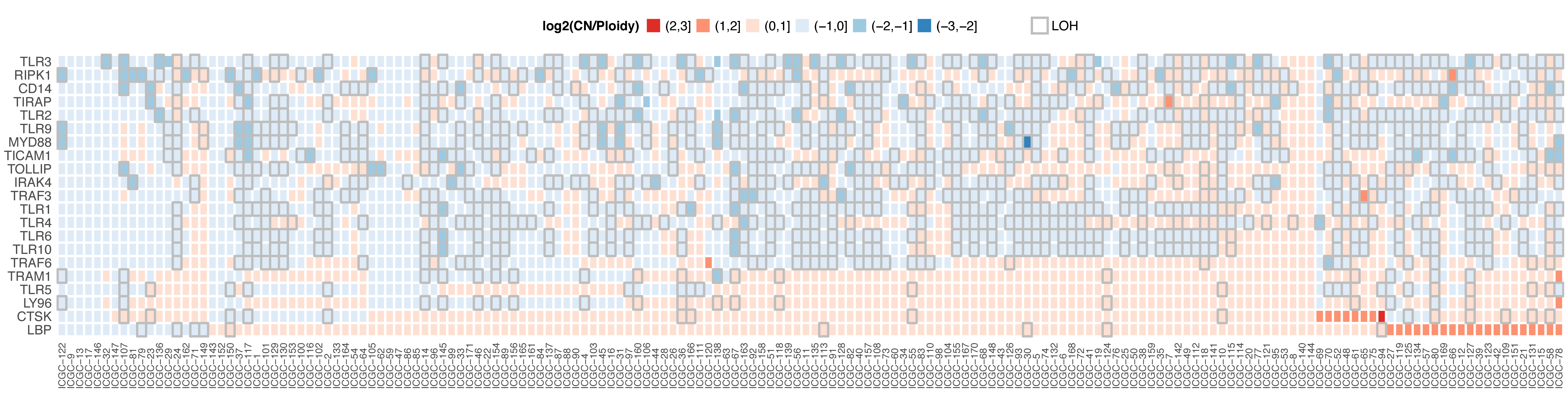

Supplement: S2 Fig — The copy number of the gene relative to the average ploidy estimated by ASCAT is shown. LOH = loss of heterozygosity. LY96 is the gene encoding MD2 protein. (TIF) [file pgen.1006808.s012.tif]

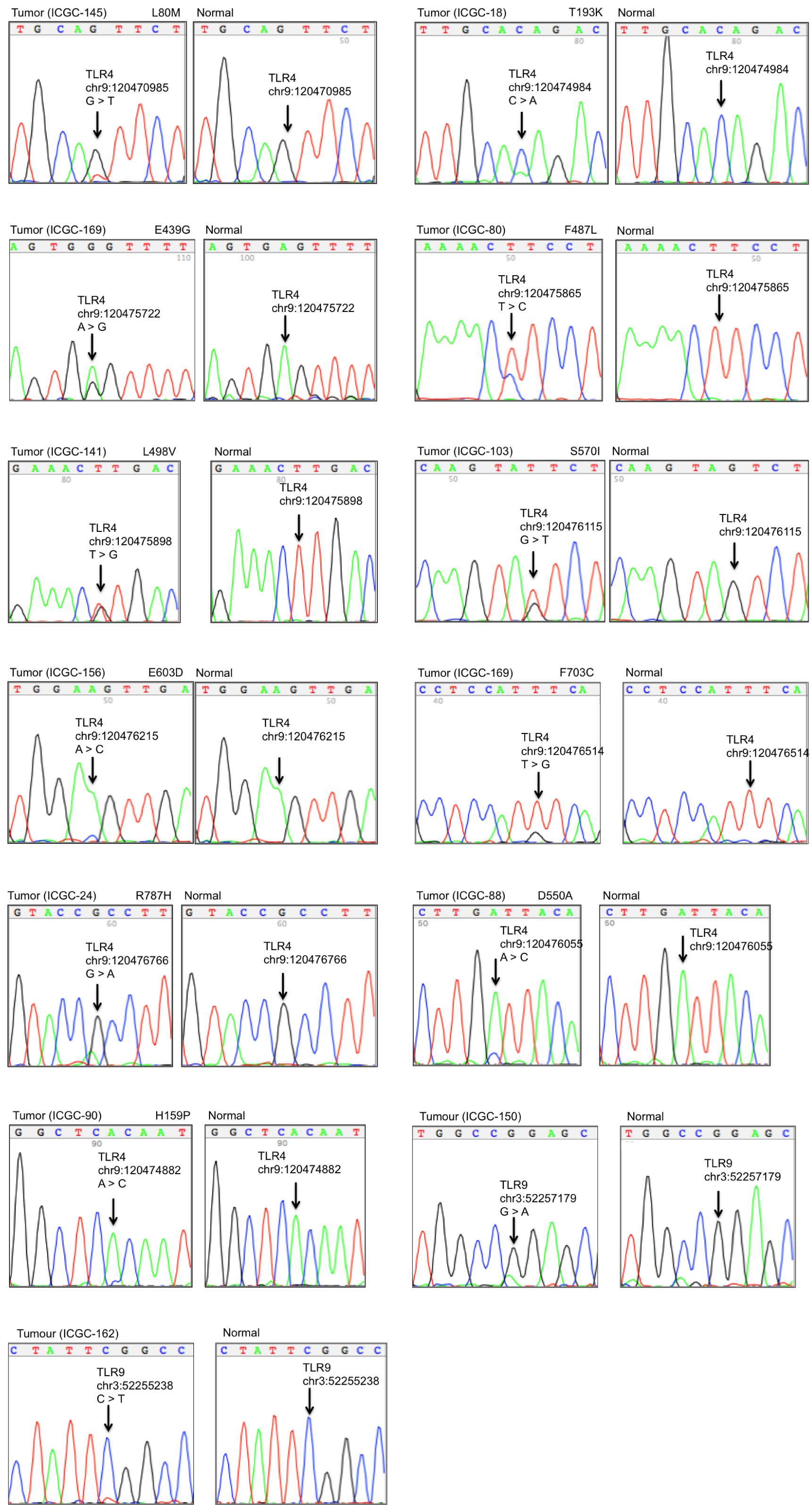

Supplement: S3 Fig — Tracings are shown for tumor and matched normal germline. Nucleotide change is in the format reference base > mutant base. (TIF) [file pgen.1006808.s013.tif]

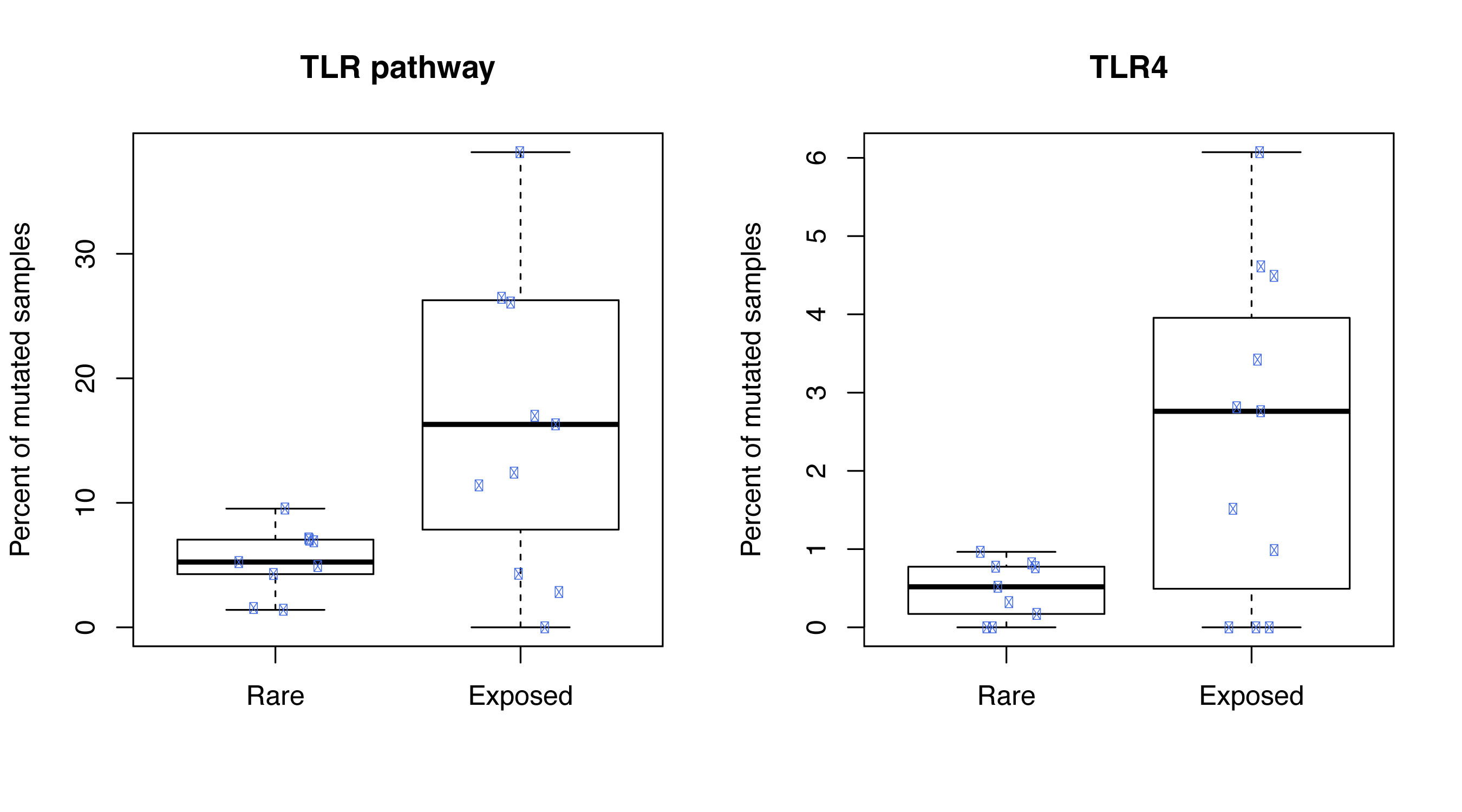

Supplement: S4 Fig — There is an increase in the frequency of mutated samples in cancer types that are highly exposed to microbes for TLR pathway mutant samples (p = 0.019, Wilcoxon rank-sum test) and TLR4 mutant samples (p = 0.028). (TIF) [file pgen.1006808.s014.tif]

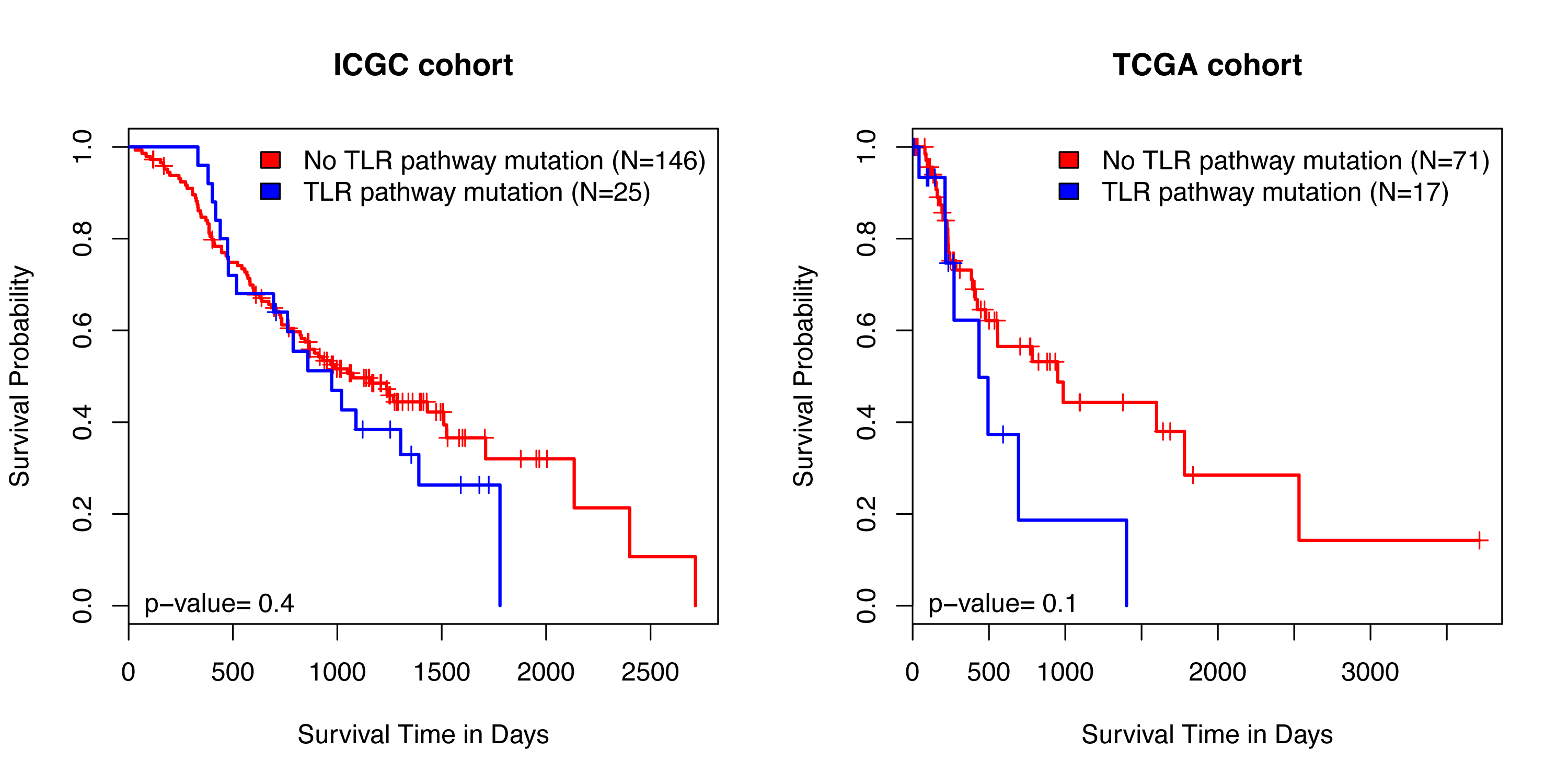

Supplement: S5 Fig — (TIF) [file pgen.1006808.s015.tif]

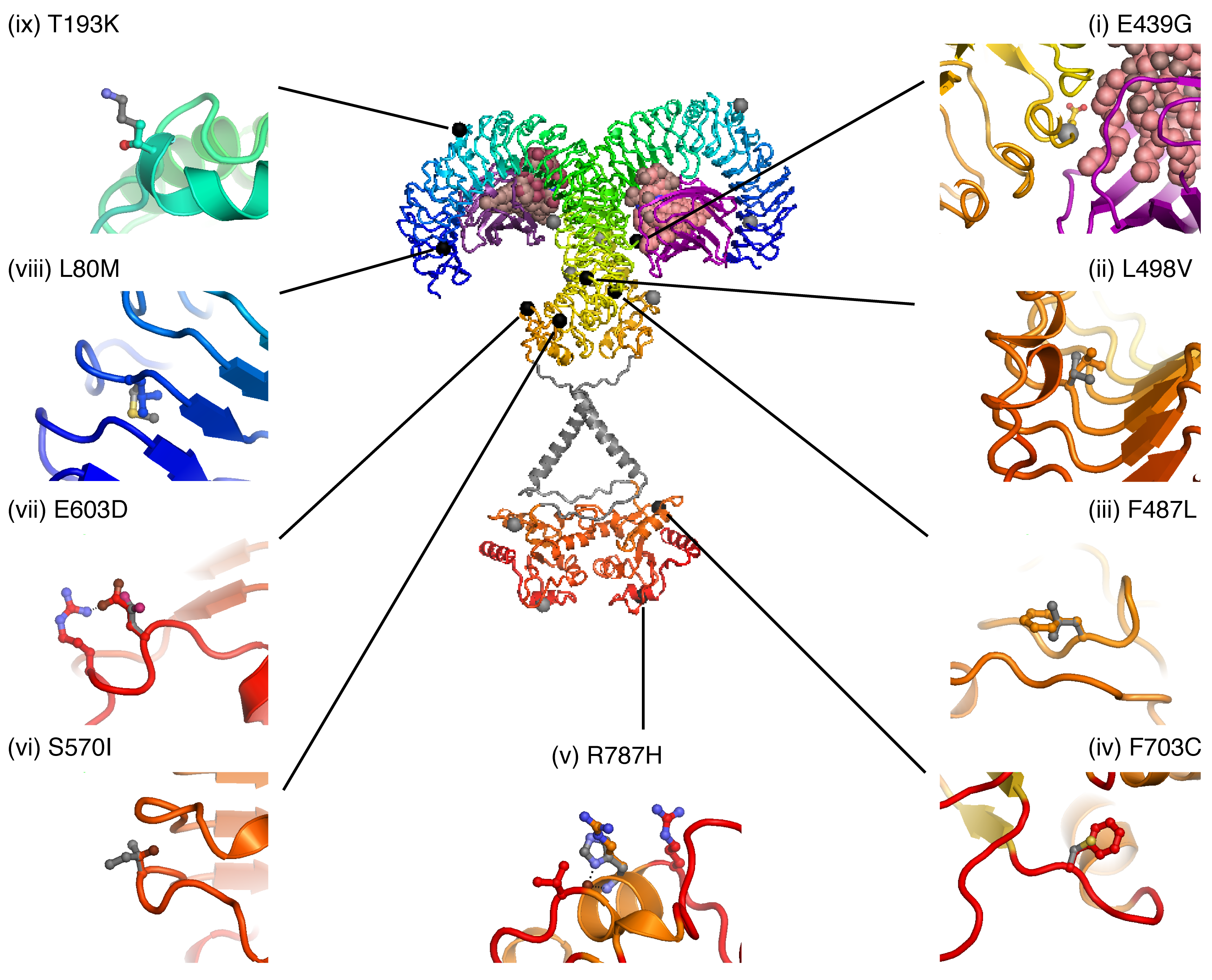

Supplement: S7 Fig — The crystal structure of dimerised human TLR4 ectodomain (colored blue to orange, PDB ID: 4G8A [22]) has been linked by transmembrane alpha helices (grey) to a hypothetical TIR domain (colored orange to red) dimer based on the known crystal structure for TLR10 (PDB ID: 2J67 [23]). MD2 is colored purple and the atoms of the bound LPS are pink. Oxygen atoms are colored pink, and nitrogen atoms are blue. Black and grey spheres indicate the TLR4 mutations on opposite sides of the TLR4 molecule. In detailed views (i) to (ix) the mutated sidechain atoms are distinguished by color (unmutated sidechain carbon atoms are in domain colors; mutated sidechain atoms are in standard atom colors, carbon gray). (TIF) [file pgen.1006808.s017.tif]

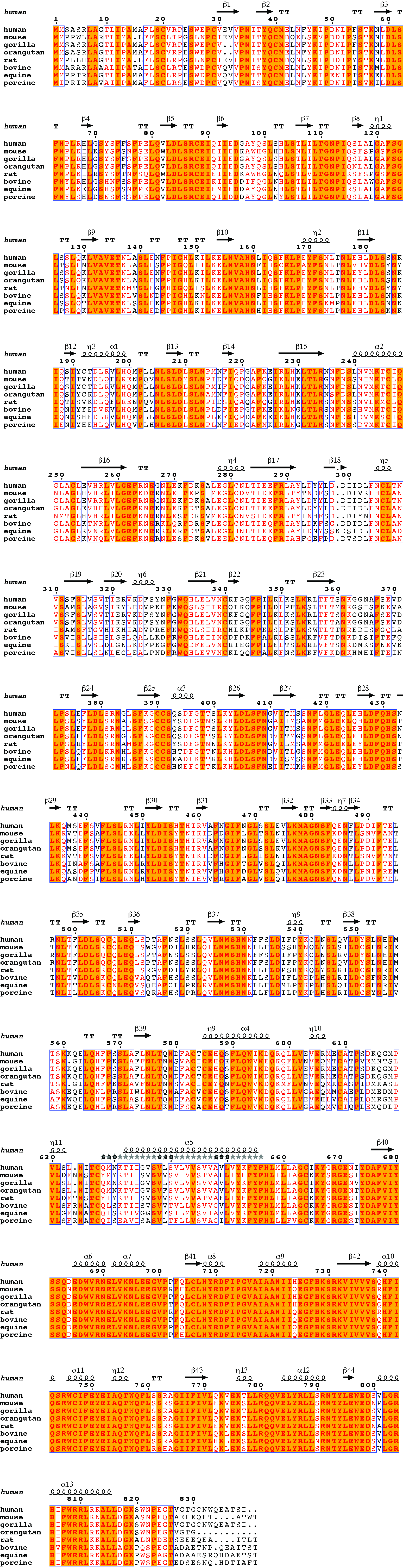

Supplement: S8 Fig — Global sequence alignment to seven non-human species (mouse, gorilla, orangutan, rat, bovine, equine and porcine) was performed using Uniprot (http://www.uniprot.org/align/) and Espript (http://espript.ibcp.fr/ESPript/cgi-bin/ESPript.cgi). Evolutionarily conserved amino acids are highlighted in orange with red text, semi-conserved amino acids have red text only, and non-conserved amino acids have black text only. The secondary structure annotation is from the model in S7 Fig (PDB ID: 4G8A [22] and PDB ID: 2J67 [23]). (TIF) [file pgen.1006808.s018.tif]

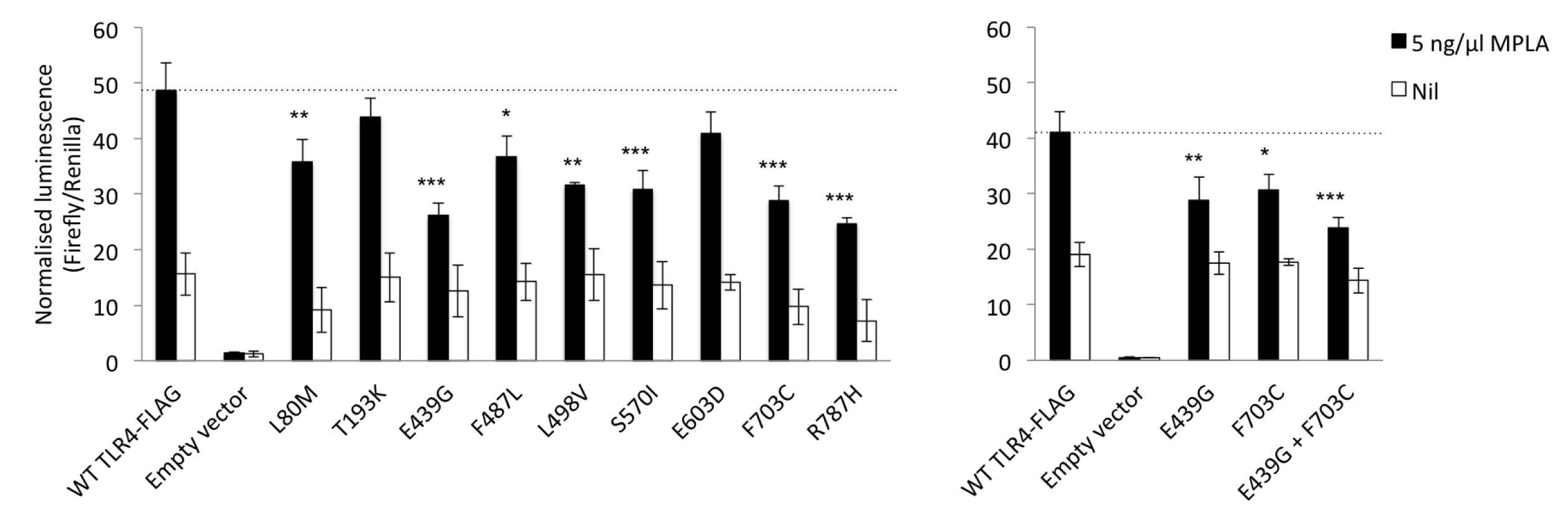

Supplement: S9 Fig — HEK293 cells were stimulated with 5 ng/μl synthetic monophosphoryl Lipid A (MPLA). The y-axis is normalized luminescence, calculated by dividing NF-κB firefly luciferase by the housekeeper renilla luciferase. Results are the average of three experiments with all conditions performed in triplicate. Error bars are standard deviation. *p<0.05, **p<0.01, ***p<0.001. (TIF) [file pgen.1006808.s019.tif]

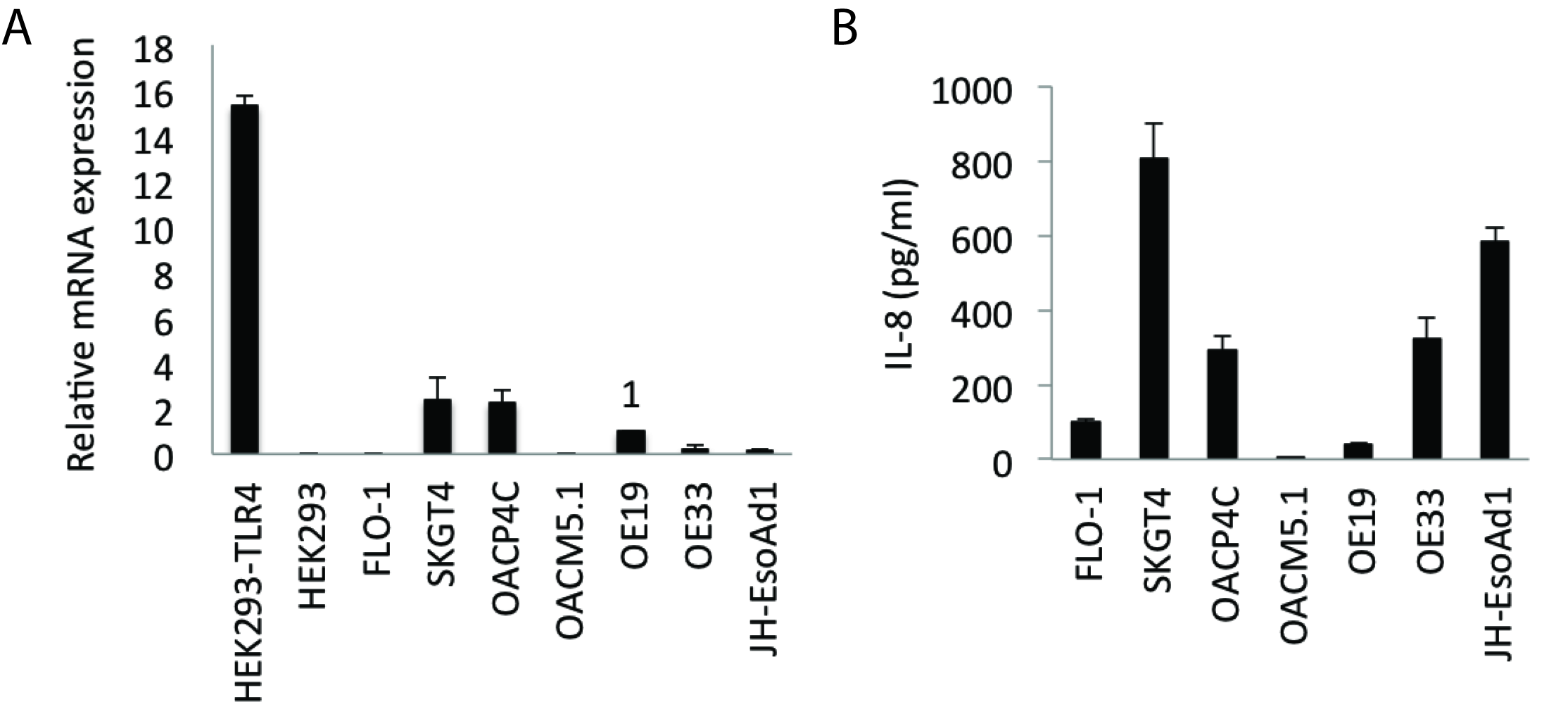

Supplement: S10 Fig — (A) Endogenous levels of TLR4 mRNA expression quantified using qRT-PCR in seven EAC lines, positive control HEK293-hTLR4-MD2-CD14 cells and negative control HEK293 cells. Relative mRNA expression is calculated as fold change relative to OE19 (defined as 1). Zero indicates an undetectable level of TLR4 (cycle threshold value greater than 35). Data are averages of three independent experiments. (B) ELISA for endogenous secretion of IL-8 in seven EAC lines, in the absence of any stimulation. Data are averages of two independent experiments, each performed in technical duplicate. Error bars are standard deviation. (TIF) [file pgen.1006808.s020.tif]

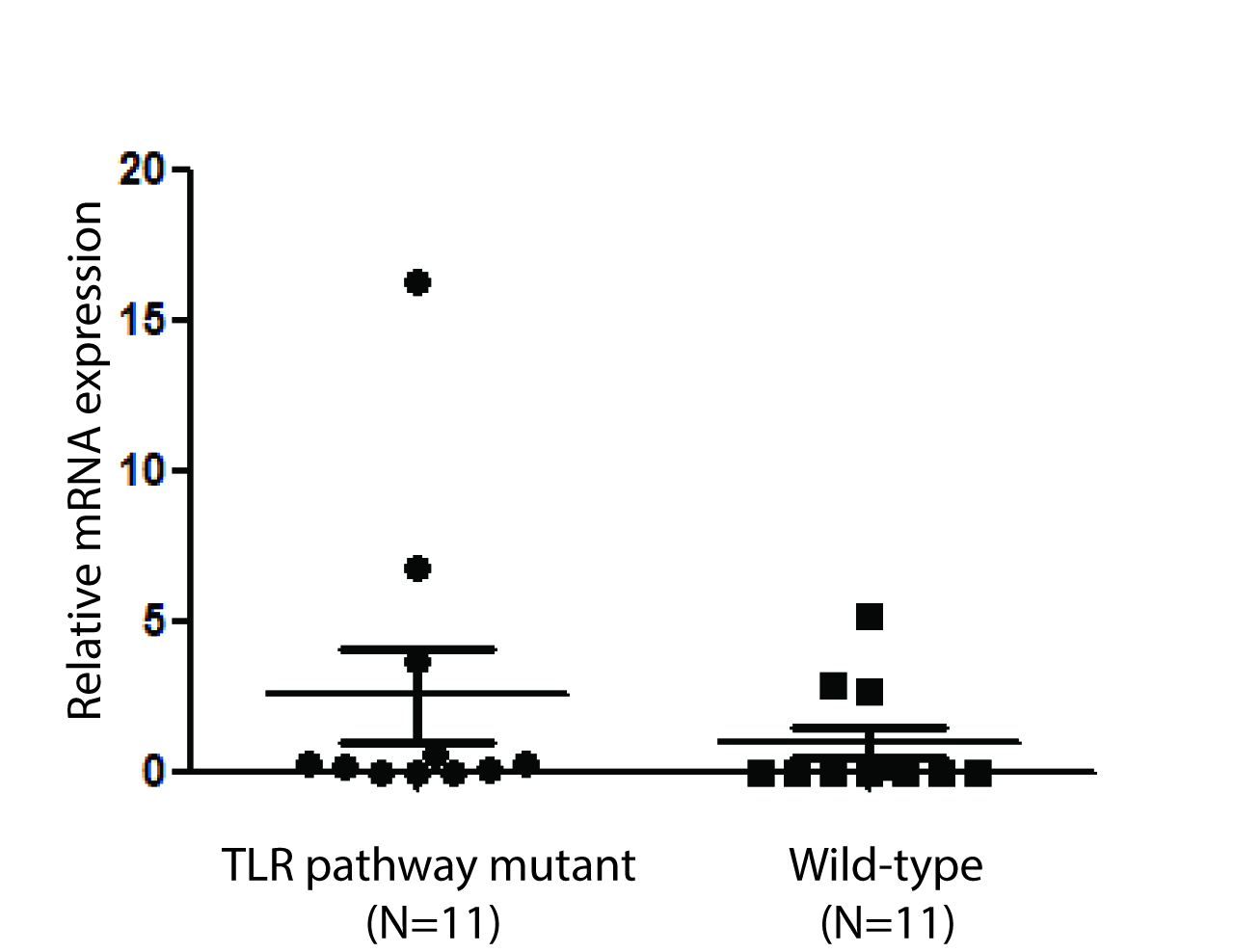

Supplement: S11 Fig — Data are an average of three technical replicates. (TIF) [file pgen.1006808.s021.tif]
